# Interaction of the Anti-Proliferative GPER Inverse Agonist ERα17p with the Breast Cancer Cell Plasma Membrane: From Biophysics to Biology

**DOI:** 10.3390/cells9020447

**Published:** 2020-02-15

**Authors:** Michaël Trichet, Rosamaria Lappano, Mathilde Belnou, Lilian Shadai Salazar Vazquez, Isabel Alves, Delphine Ravault, Sandrine Sagan, Lucie Khemtemourian, Marcello Maggiolini, Yves Jacquot

**Affiliations:** 1 Institut de Biologie Paris-Seine (IBPS), Service de Microscopie éLectronique (IBPS-SME), Sorbonne Université, CNRS, 75005 Paris, France; michael.trichet@sorbonne-universite.fr; 2 Department of Pharmacy, Health and Nutritional Sciences, University of Calabria, 87036 Rende, Italy; lappanorosamaria@yahoo.it; 3 Laboratoire des Biomolécules, LBM, CNRS UMR 7203, Sorbonne Université, Ecole Normale Supérieure, PSL University, 75005 Paris, France; mathilde.belnou@free.fr (M.B.); shadai.salazar.v@gmail.com (L.S.S.V.); delphine.ravault@sorbonne-universite.fr (D.R.); sandrine.sagan@sorbonne-universite.fr (S.S.); lucie.khemtemourian@u-bordeaux.fr (L.K.); 4 Institute of Chemistry & Biology of Membranes & Nanoobjects (CBMN), CNRS UMR 5248, Université de Bordeaux, Institut Polytechnique Bordeaux, 33600 Pessac, France; i.alves@cbmn.u-bordeaux.fr; 5 Cibles Thérapeutiques et Conception de Médicaments (CiTCoM), CNRS UMR 8038, U1268 INSERM, Faculté des Sciences Pharmaceutiques et Biologiques, Université Paris Descartes, 75006 Paris, France

**Keywords:** apoptotic peptide, lipid vesicles, breast cancer cells, aggregates, membrane interaction, internalization

## Abstract

The peptide ERα17p, which corresponds to the 295-311 fragment of the hinge/AF2 domains of the human estrogen receptor α (ERα), exerts apoptosis in breast cancer cells through a mechanism involving the G protein-coupled estrogen-dependent receptor GPER. Besides this receptor-mediated mechanism, we have detected a direct interaction (Kd value in the micromolar range) of this peptide with lipid vesicles mimicking the plasma membrane of eukaryotes. The reversible and not reversible pools of interacting peptide may correspond to soluble and aggregated membrane-interacting peptide populations, respectively. By using circular dichroism (CD) spectroscopy, we have shown that the interaction of the peptide with this membrane model was associated with its folding into β sheet. A slight leakage of the 5(6)-fluorescein was also observed, indicating lipid bilayer permeability. When the peptide was incubated with living breast cancer cells at the active concentration of 10 μM, aggregates were detected at the plasma membrane under the form of spheres. This insoluble pool of peptide, which seems to result from a fibrillation process, is internalized in micrometric vacuoles under the form of fibrils, without evidence of cytotoxicity, at least at the microscopic level. This study provides new information on the interaction of ERα17p with breast cancer cell membranes as well as on its mechanism of action, with respect to direct membrane effects.

## 1. Introduction

The Pro^295^–Thr^311^ sequence of the human estrogen receptor α (ERα) encompasses residues that are located in the C-terminal part of the hinge region (D domain) and in the N-terminus of the AF2 transactivation function (E/F domains). This short sequence corresponds to an interaction platform that is in charge of the recruitment of protein partners such as Ca^2+^-calmodulin [1] and Hsp70 [2]. Following the X-ray crystal structures of ERα~ligand complexes available in the Protein Data Bank (PDB), the 301-311 surface-exposed sequence is principally folded in polyproline II (PPII), an observation that is relevant to its involvement in the recruitment of protein partners [3,4]. In addition, it is subjected to post-translational modifications such as phosphorylation, acetylation, and SUMOylation (see [5] and references herein). It is also subjected to trypsin- and chymiotrypsin-dependent proteolysis at the K^303^N^304^ and at the K^302^K^303^ sites [6]. In this regard, it should be noted that the K^299^RSKK^303^ motif corresponds to the third nuclear localization signal (NLS) of the ERα [7]. Furthermore, it is located in the core of the autonomous activation function AF2a (residues 282 to 351) [8]. The ERα mutation K303R, which is found in several invasive breast tumors [9], is associated with methylation modifications by the SET7 methyltransferase and, therefore, with transcription profile changes [10]. Altogether, these observations strongly suggest that the 295-311 part of the ERα is important for the control of transcription.

In the light of these observations, we have synthesized the 17-mer peptide corresponding to the ERα 295-311 region (sequence: H_2_N-PLMIKRSKKNSLALSLT-COOH, peptide ERα17p) and we have tested its action in different experimental conditions and on different breast cancer cell lines. For unknown reasons, this peptide is estrogenic in steroid-deprived conditions [2,11,12,13,14], whereas it is anti-proliferative in complete (i.e., physiological) serum conditions, as evidenced by the decrease of the Bcl_xL/_Bax ratio, the increase of cleaved caspase-9, and changes in cell migration and actin network [15,16,17,18]. Albeit much more weakly than in cells expressing the ERα, apoptosis is also observed in ERα-negative breast cancer cell lines, suggesting the involvement of other estrogen-dependent receptor(s). We have shown that the heptatransmembrane G protein-coupled estrogen receptor (GPER) was functionally and physically targeted by the peptide, which triggers an inverse agonist action [17,19,20]. Following a modeling approach, the N-terminal PLMI motif of the peptide ERα17p seems to engulf within a cavity located in the extracellular part of the GPER [17], where other ligands such as the quinoleins G-1 and G-15 and the benzopyrroloxazine PBX-2 interact [21,22,23]. Its mechanism of action refers to a pharmacological process through which it induces a proteasome-dependent decrease of GPER protein levels and abrogates the activation of (i) the epidermal growth factor receptor (EGFR), (ii) the extracellular signal-regulated kinase ERK1/2, and (iii) the transcription factor c-fos [17]. The ability of ERα17p to hinder the GPER-mediated signal could explain its anti-proliferative action and its capacity to decrease by ~50% the volume of xenografted triple negative breast cancer (TNBC), an aggressive tumor type, with a low dose of ERα17p (i.e., 1.5 mg/kg, three times per week during four weeks) [15,17,24].

By using circular dichroism (CD) spectroscopy and differential scanning calorimetry (DSC), we have previously shown that the peptide ERα17p was able to interact with membrane models (LUVs) that are composed of one species of anionic lipids such as 1,2-dimyristoyl-sn-glycero-3-phosphoglycerol (DMPG) [25]. A β-sheet secondary structure and membrane leakage are the hallmarks of this interaction [25]. In the presence of zwitterionic lipids such as 1,2-dimyristoyl-sn-glycero-3-phosphocholine (DMPC), no interaction is observed [25]. Although hydrophobic contributions seem likely, electrostatic interactions appear as the driving force of this lipid-binding process. It is of note that at high concentrations (> 50 μM) or in an anionic environment different from anionic lipids (e.g., pH > 7, mica substrate [26]), ERα17p forms amyloid-like fibrils, which are also characterized by a β-sheet secondary structure [25,26]. This raises the question of whether such interaction, which could be related to the formation of fibrils and, therefore, of aggregates, can also be observed with eukaryotic cells and if so, whether it could cause cell death through direct alterations at the cytoplasmic membrane (e.g., formation of pores).

To answer this question, we first investigated whether the interaction of the peptide ERα17p with lipids could also occur with an eukaryote plasma membrane model composed of a mixture of POPC (1-palmitoyl-2-oleoyl-sn-glycero-3-phosphocholine), POPS (1-palmitoyl-2-oleoyl-sn-glycero-3- phospho-l-serine), and Chol (cholesterol) at a ratio 7:1:2 [27,28,29,30]. To this aim, we used far-UV circular dichroism (CD), plasmon waveguide resonance (PWR) and fluorescence (leakage assay) spectroscopy techniques. Then, we extended our study to living MCF-7 breast cancer cells and we explored the effects of the peptide on the cellular morphology, with a special emphasis on the cytoplasmic membrane. By using different microscopy techniques, we observed ERα17p aggregates at the plasma membrane. These aggregates and their fate, with respect to internalization, were studied using scanning (SEM) and transmission (TEM) electron microscopy. At least, we explored the possibility of direct cell membrane cytotoxic effects from ERα17p by using trypan blue and CCK8 (WST-8) assays. The data reported here provide new insights on the mechanism of action of the GPER modulator ERα17p and on its ability to promote direct alterations at the cell membrane that would be responsible for cell death.

## 2. Materials and Methods

### 2.1. Materials and Cell Cultures

1-palmitoyl-2-oleoyl-sn-glycero-3-phosphocholine (POPC) and 1-palmitoyl-2-oleoyl-sn- glycero-3-phospho-l-serine sodium salt (POPS) were purchased from Avanti Polar Lipids (Alabaster, AL, USA). Cholesterol (Chol) was ordered at Genzyme Pharmaceuticals (Baar, Switzerland). 5(6)-carboxyfluorescein was purchased from Sigma-Aldrich (Saint-Quentin Fallavier, France). The peptide ERα17p was obtained and characterized as previously described [14,25]. MCF-7 breast cancer cells were cultured in growth Dulbecco’s Modified Eagle Medium DMEM (ThermoFisher Scientific, Courtaboeuf, France) supplemented with fetal calf serum (FCS), penicillin/streptomycin (100,000 IU/L), and amphotericin B (1 mg/L), in a humidified atmosphere containing 5% CO_2_ at 37  °C.

### 2.2. Preparation of Large Unilamellar Vesicles (LUV)

Large unilamellar vesicles (LUV) of POPC/POPS/Chol (ratio 7:1:2, mol/mol/mol) were prepared from mother solutions of lipids at a concentration of 5 µM in chloroform, as already described [25]. The chloroform was eliminated under a gentle stream of gaseous nitrogen and then placed under vacuum during 30 min. Five freeze–thaw cycles were carried out by alternately placing the sample vials in a liquid nitrogen bath and a warm water bath. The mixture was extruded twenty times through a 200 nm polycarbonate Nucleopore membrane filter by using a mini-extruder (Avanti Polar Lipids, Alabaster, AL, USA). Phosphorus concentrations of the LUV solutions were measured by using the method published by Rouser et al. [31].

### 2.3. Far-UV Circular Dichroism (CD) Spectroscopy

Far-UV circular dichroism was performed on a Jasco 810 spectropolarimeter (Jasco Inc., Easton, MD, USA) equipped with a Peltier. Spectra were recorded over the 190–260 nm wavelength range by using a 0.1 cm pathlength quartz cell. CD spectra were recorded as an average of four scans and at a temperature of 25 °C with 0.2 nm steps and a 10 nm.min^−1^ scan speed. A 25 µM ERα17p solution was added to 250 µM of POPC/POPS/Chol (ratio 7:1:2, mol/mol/mol) in 10 mM phosphate buffer, pH 7.5. Data are presented in mean residue ellipticity (Δθ, in deg. cm^2^. dmol^−1^. resid^−1^) as a function of the wavelength (in nm).

### 2.4. Plasmon Waveguide Resonance (PWR)

Self-assembled lipid bilayers used for PWR experiments were obtained from a 10 mg/mL solution of POPC/POPS/Chol in a butanol/squalene mixture (0.93:0.07, *v*/*v*). PWR assays were performed by using a custom apparatus with increased performance [32] when compared to the prototype designed by the group of Tollin [33]. Light was generated from a polarized CW laser (He-Ne; wavelengths of 632.8 and 543.5 nm) incident on the back surface of a thin metal film (Ag) deposited on a glass prism and coated with a layer of SiO_2_ [33,34]. The spectral angular resolution was ≤ 1 mdeg. PWR spectra corresponded to plots of reflected light intensity versus incident angle. *S*-polarized (*s*-pol) spectra is obtained when the electric vector is parallel to the plane of the resonator surface and *p*-polarized (*p*-pol) light, when the vector is perpendicular to such a plane. A protocol initially developed by Mueller et al. was used to make black lipid membranes across a small hole in a Teflon block [35]. Briefly, a small amount of lipid solution was injected into the orifice of a Teflon block separating the silica surface of the PWR resonator from the aqueous phase [34]. The spontaneous bilayer formation was initiated when the sample compartment was filled with aqueous buffer solution [33]. After the stabilization of the lipid bilayer (no further spectral changes with time), the peptide ERα17p was incrementally added to the PWR cell sample. Spectral changes were acquired for both polarizations. The system was let to equilibrate before each peptide addition. PWR being sensitive to the optical properties of material deposited on the resonator surface (so peptide bound to the lipid bilayer) means that interference from the material present in the bulk solution (non-bound) is unlikely. Apparent dissociation constants (Kd, in μM) were obtained by plotting the resonance minimum position as a function of the peptide concentration and by fitting the plot through a hyperbolic binding function using GraphPad Prism™ version 5.0a (GraphPad Software, San Diego, California, USA). Kinetic experiments were calculated from changes in the resonance minimum position at specific peptide concentrations and as a function of time. Data fitting was performed with a one-stage exponential association equation using GraphPad Prism™.

### 2.5. Membrane Permeability Assay in Large Unilamellar Vesicles

The change of fluorescence of 5(6)-carboxyfluorescein was used to monitor the membrane permeabilization induced by the peptide ERα17p. Before the five freeze–thaw cycles required for forming LUVs (see #2.2), the lipid film was completely rehydrated for 30 min with a 70 mM solution of 5(6)-carboxyfluorescein. The removal of free 5(6)-carboxyfluorescein was carried out by a Sephadex G50 gel filtration. The leakage of the 5(6)-carboxyfluorescein was detected in a 96 wells transparent microtiter plate by using a Fluostar OPTIMA plate reader (BMG Labetch, Champigny s/Marne, France) at excitation and emission wavelengths of 485 and 520 nm, respectively. The final concentrations were 100 mM or 200 mM for lipids, and 10 mM for the peptide (peptide:lipid ratio of 1:10 and 1:20, respectively). Directly after addition of all components, the microtiter plate was shaken for 10 s using the shaking function of the plate reader. The plate was not shaken during the measurements. To delimit the assay endpoint, we used 10% Triton X-100 during 12 h, approximately, after starting the 5(6)-carboxyfluorescein measurements. The maximum leakage at the end of each measurement was determined via the addition of 1 mL of 10% Triton X-100 to a final percentage of 0.05% (*v*/*v*). The release of fluorescent dye was normalized according to the following equation:

L(t) = (F_t_ − F_0_)/(F_100_ − F_0_)

where L(t) is the fraction of released 5(6)-carboxyfluorescein; F_t_ the fluorescence intensity measured at a time; and where F_0_ and F_100_ are the fluorescence intensities at t = 0 and at the endpoint, respectively. Two independent experiments were performed in triplicate.

### 2.6. Scanning (SEM) and Transmission (TEM) Electron Microscopy

MCF7 cells were cultured in Dulbecco’s Modified Eagle Medium (DMEM) glutamax supplemented with 10% fetal calf serum (FCS). A total of 50,000 cells were cultured, a day before, on glass cover slips in 96-well plates. After 24 h, cells were 40% confluent. After washing steps with the culture medium, cells were incubated for 48 h with 10 μM ERα17p in culture medium and at a temperature of 37 °C. After 24 h of incubation, the medium and the peptide were renewed. Cells were then washed three times with cold medium. Then, they were washed with 100 mM phosphate buffer (pH 7.4), and fixed with 2% glutaraldehyde in 0.1 M phosphate buffer pH 7.4 for 1 h at room temperature. Glass cover slips were stored at 4 °C. Samples were extensively washed with 0.1 M cacodylate buffer solution and incubated for 20 min (SEM) or 1 h (TEM) in 1% osmium tetroxide dissolved in 0.1 M cacodylate buffer, and dehydrated through graded concentrations of ethanol.

For the SEM experiments, the cells were critical point dried (CPD300, Leica microsystems, Nanterre, France), mounted on platinum-sputtered stubs (ACE600, Leica microsystems, Nanterre, France), and observed with a field-emission SEM operating at 3 kV (GeminiSEM500, Zeiss, Marly le Roi, France). Secondary electrons were collected with an in-lens detector. Current, scan speed, and line integration were adjusted during observation.

For the TEM experiments, dehydration was continued with anhydrous acetone before progressive embedding in epoxy resin (Agar 100 resin kit, Agar Scientific, Stansted, UK). Sections were then cut into ultra-thin 70 nm slices (Ultracut, Leica Microsystems, Nanterre, France), collected on copper grids, and double-stained with 2.5% aqueous uranyl acetate and 1% lead citrate.

For the negative contrast of fibrils (TEM), aliquots (20 μL) of ERα17p at a concentration of 100 μM in 0.2 M PBS and at pH 7.4 were studied. After 1 h of incubation, the samples were adsorbed onto glow-discharged carbon-coated 300-mesh copper grids for 2 min. Grids were negatively stained for 45 s on 2.5% uranyl acetate and were then blotted and dried. TEM observations were achieved at 80 or 200 kV with a JEOL2100HC (Croissy, France) equipped with a side-mounted 2k × 2k Veleta CCD camera (Olympus, Rungis, France) and a post-column Gatan Image Filter (GIF Tridiem, Grandchamp, France).

### 2.7. Cytotoxicity Assays

ERα17p effects on MCF-7 cell viability were evaluated with trypan blue and CCK8 (Dojindo Molecular Technologies, Inc., Le Perray-en-Yveline, France) assays. A total of 20,000 cells were seeded in 96-multi-well plates 24 h before the assays. The culture medium (DMEM glutamax, 10% FCS) was replaced by a fresh one containing 1 or 10 μM ERα17p for 1 or 48 h incubation. After an incubation time of 24 h at 37 °C, the medium containing ERα17p was renewed and incubated for additional 24 h. After peptide incubation, cells were then washed with Hank’s balanced salt solution (HBSS) and further incubated with 10% CCK8 in complete culture medium. After 2 h of incubation at 37 °C, OD (450 nm and 620 nm for the reference) was measured (PolarStar, BMG Labtech). Alternatively, after peptide incubation, cells were detached with 20 μL trypsin. Then, trypsin digestion was stopped by the addition of 80 μL DMEM with 10% FCS. A mixture of cells (10 μL) with 10 μL trypan blue was then prepared to determine the presence of particles and viable cells using the automated cell counter Countess II (Thermofisher, Illkirch, France).

## 3. Results and Discussion

### 3.1. ERα17p Interacts with Lipidic Bilayers Mimicking the Eukaryote Plasma Membrane and Alters Their Integrity

Using one type of phospholipid, only, we have previously shown that the peptide ERα17p was prone to interact in its full length with negatively charged vesicles and micelles, through a mechanism implying non-specific electrostatic and hydrophobic forces [25,36]. Here, we have used vesicles composed of 1-palmitoyl-2-oleoyl-sn-glycero-3-phosphocholine (POPC), 1-palmitoyl-2-oleoyl-sn-glycero-3-phospho-l-serine (POPS), and cholesterol (Chol), with a ratio of 7:1:2 (mol/mol/mol), respectively. Such, vesicles are prone to mimic the plasma membrane of eukaryotes [27,28,29,30,37]. Far-UV circular dichroism (CD) spectroscopy, plasmon waveguide resonance (PWR) and fluorescein leakage experiments were carried out to explore the secondary structure of the peptide under membrane interaction, the characteristics of this interaction, and the effects of ERα17p on the membrane integrity, respectively.

#### 3.1.1. Far-UV Circular Dichroism (CD)

CD spectroscopy allows for access to peptide conformational changes in the presence of lipids. Thus, we have used this technique to reach information on the ERα17p secondary structure in the presence of POPC/POPS/Chol [25,38]. It has been previously shown that ERα17p adopts a β-sheet secondary structure (typical positive and negative bands of similar intensity at about 200 and 220 nm, respectively [39]), when in contact with purely anionic lipids [25]. With zwitterionic (neutral) phospholipids, it remains random coil (strong negative band at ~200 nm), as also observed when alone in solution [12,25]. Thus, the β-sheet CD spectrum signature reflects membrane interaction, whereas a random coil conformational state means a lack of interaction [25].

**Figure 1:**
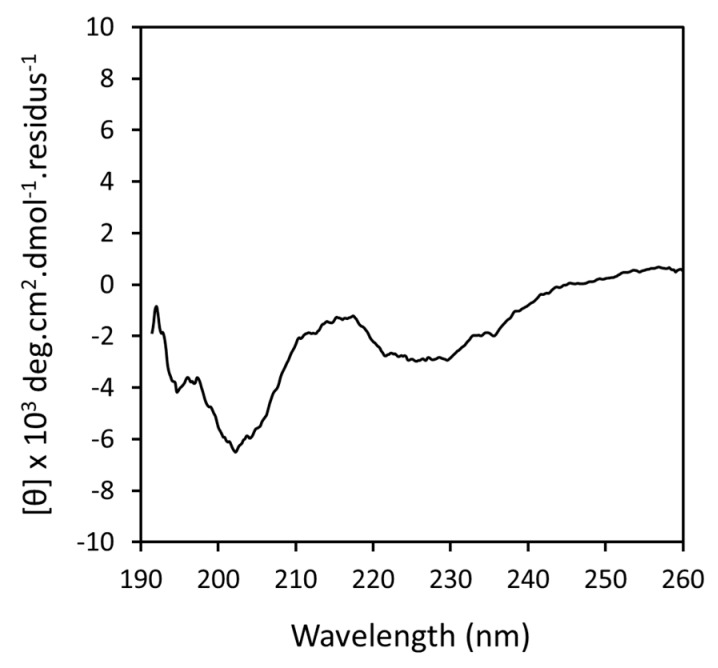
ERα17p Circular Dicroism (CD) spectrum recorded between 190 and 260 nm with a peptide concentration of 25 µM and in the presence of 250 µM of POPC/POPS/Chol (7:1:2 mol/mol/mol) in 10 mM phosphate buffer (pH 7.5) and at a temperature of 25 °C (incubation time: 13 h). The mean residue ellipticity Δθ (in deg.cm^2^.dmol^−1^.cm^−1^.resid.^−1^) is expressed as a function of the wavelength (in nm).

In the presence of POPC/POPS/Chol (ratio 7:1:2 mol/mol/mol, respectively) and in phosphate buffer (pH 7.5), the ERα17p CD spectrum profile shows two negative maxima, at ~200 and 230 nm (Figure 1). Whereas the former could be relevant to a random coil peptide population, the second band recorded at ~230 nm (n → π* electronic transition [40]) is rather relevant to the β-sheet (i.e., to an interacting peptide population) [25]. The weak intensity of the negative signal at ~200 nm results not only from a strong negative signal corresponding to the random state of the peptide, but also from the presence of the π → π* electronic transition (positive signal) of the β-sheet signature, which occurs at the same wavelength region. It is of note that similar results have already been obtained with amyloid β (11–28)-derived peptides [41]. Referring to the signal recorded at ~230 nm, approximately 50% of the peptide amount seems to be in β-sheet, contrasting with purely anionic vesicles, where 100% of the peptide amount seems to interact (automated deconvolution of the CD spectra using PepFit algorithm [42]). These results are not only in agreement with those results previously reported with DMPC and DMPG [25], but also highlight the importance of negative charges in this interaction.

#### 3.1.2. Plasmon Waveguide Resonance (PWR)

**Figure 2:**
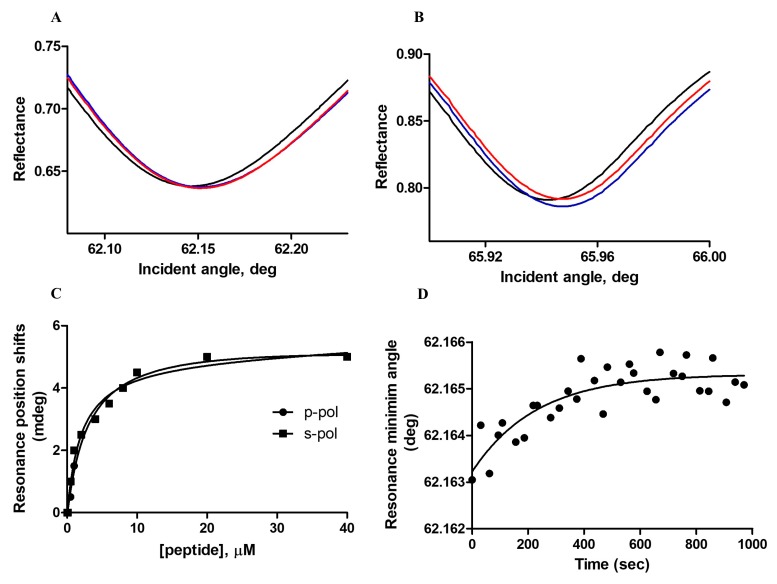
ERα17p interaction with POPC/POPS/Chol membrane followed by Plasmon Waveguide Resonance (PWR) in an aqueous buffer solution at a temperature of 25 °C. PWR spectra corresponding to the bilayer alone (black), the addition of saturating concentrations of peptide (blue) and the wash of the lipid bilayer with buffer (red) are shown for (**A**) the *p*- and (**B**) the *s*-polarizations. (**C**) Binding curve obtained from the shifts in the minimum resonance angle (directly provided by the instrument software) obtained for both *p*- and *s*-polarizations at equilibrium upon each incremental peptide addition. (**D**) Kinetic data obtained with the *p*-polarization after the addition of 30 μM of peptide.

To obtain further details concerning the mode of binding of the peptide ERα17p with the tested phospholipidic mixture, plasmon waveguide resonance (PWR) was carried out. At saturating peptide concentration, positive shifts (5 ± 1 mdeg) for both the *p*- and *s*-polarizations were observed, indicating not only an interaction but also a reorganization of the lipids (Figure 2A,B and Table 1). The similarity of the two shift values highly suggests a lack of predominant orientation of the peptide within the membrane and an anisotropic lipidic rearrangement. A Kd of 1.2 ± 0.3 μM was calculated after titration (Figure 2C). Importantly, the addition of a saturating peptide concentration followed by a wash step of the chamber with the buffer solution was responsible for a negative shift recovery in the resonance minimum position and, therefore, for the removal of the peptide. As this process was not completely reversible, a buried pool of peptide at the membrane should be evoked.

The kinetics of the association of the peptide with POPC/POPS/Chol was monitored by recording changes at the minimum resonance position for both *p*- and *s*-polarization orientations, as a function of time (Figure 2D). This process was explored for peptide concentrations close to the affinity constant value (i.e., close to equilibrium). Rate constants were similar for both orientations, confirming the absence of the preferred peptide orientation (Table 1).

#### 3.1.3. Leakage Experiments

**Figure 3:**
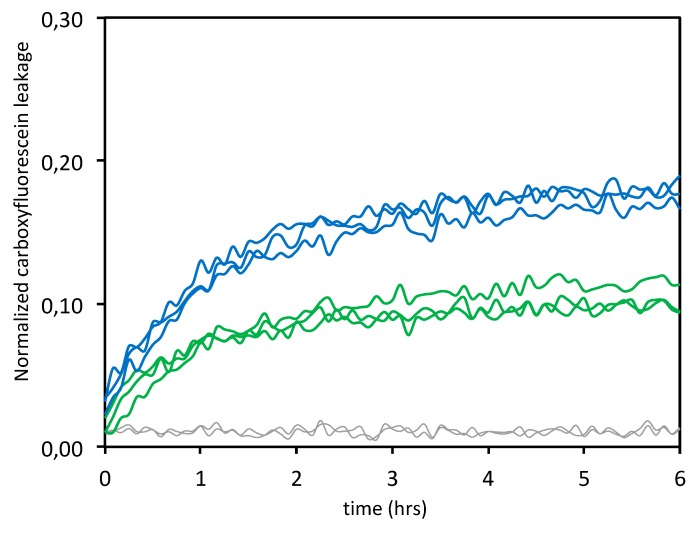
ERα17p-induced 5(6)-carboxyfluorescein leakage observed by fluorescence spectroscopy in the presence of POPC/POPS/Chol 7:1:2. The peptide at 10 μM was added to the 5(6)-carboxyfluorecscein solution containing large, unilamellar vesicles (LUVs) composed of POPC/POPS/Chol at a lipid:peptide ratio of 10:1 (blue lines) and 20:1 (green lines), at t = 0. The maximum leakage, after complete disruption of all vesicles by Triton X-100, was set at 1. The normalized fluorescence signal of the vesicles in the absence of the peptide is represented in grey. All spectra are recorded in a phosphate buffer solution (pH 7.4) over 6 h and at 25 °C (*n* = 2).

Leakage experiments were carried out in phosphate buffer and at physiological pH, at two different peptide:lipid ratios (i.e., 1:10 and 1:20). Upon addition of a membrane-damaging peptide, disruption of the lipid bilayer allows the escape of the fluorophore (here, 5(6)-carboxyfluorescein) and eliminates, therefore, the self-quenching. An increase in the fluorescence signal is consequently recorded [43]. Our data indicate that the ERα17p used at the biologically active concentration of 10 μM [11,12,14] with POPC/POPS/Chol (7:1:2 mol/mol/mol) induces about 18% membrane leakage at the lipid:peptide ratio of 10:1 and around 10% at the lipid:peptide ratio 20:1 (Figure 3). A two-fold decrease of the percentage of membrane leakage is found, as previously published with vesicles composed of anionic lipids, exclusively [25].

For both ratios, a rapid and near-exponential increase of the fluorescence signal was recorded, suggesting transient pore formation at the membrane, as usually found with antimicrobial peptides [44,45]. No leakage was observed in the absence of the peptide (control). These data are clearly in agreement with previous results, as they reveal that the peptide ERα17p induces a concentration-dependent leakage of the 5(6)-carboxyfluorescein with vesicles containing negatively charged lipids [25]. When compared with purely negative DOPG vesicles (45% membrane leakage with a lipid:peptide ratio 10:1 and 20% membrane leakage with a lipid:peptide ratio 20:1, see [25] for more details), the dramatic decrease in membrane leakage recorded here could result from lower amounts of surface-exposed negative charges. These results are in agreement with the above CD results (see #3.1.1.).

By using 10% phosphatidylserine, we have confirmed the importance of negatively charged lipids in the interaction of ERα17p with LUVs [25]. The fact that phosphatidylserine, which is abundant in the outer membrane leaflet of human tumor cells (10 to 15% of total lipids [46,47,48,49]), harbors a carboxylate group in its polar head, explains this interaction and the related leakage. Now, the question arises as to whether membrane alterations could also be observed in cancer cells and, therefore, if it could participate in the anti-proliferative action of the peptide ERα17p.

### 3.2. The ERα17p Forms Peptide Aggregates Interacting with Plasma Membrane

#### 3.2.1. SEM and TEM *Experiments*

**Figure 4:**
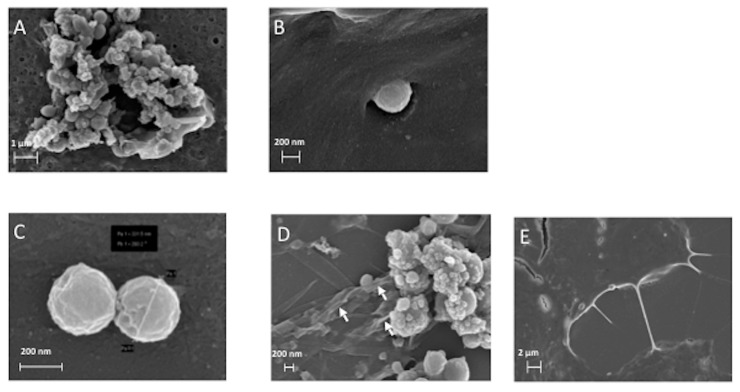
Scanning electron microscopy (SEM) pictures of: (**A**,**B**) the peptide ERα17p at the active concentration of 10 μM after 48 h incubation with MCF-7 cells; (**C**,**D**) the peptide ERα17p, alone, at a concentration of 10 μM; (**E**) MCF-7 cells incubated in the absence of peptide (control). The white arrows in (**D**) show peptide fibrils. All experiments were carried out over 48 h and in DMEM. Mean diameter of MCF-7 cells: 22.96 μm.

**Figure 5:**
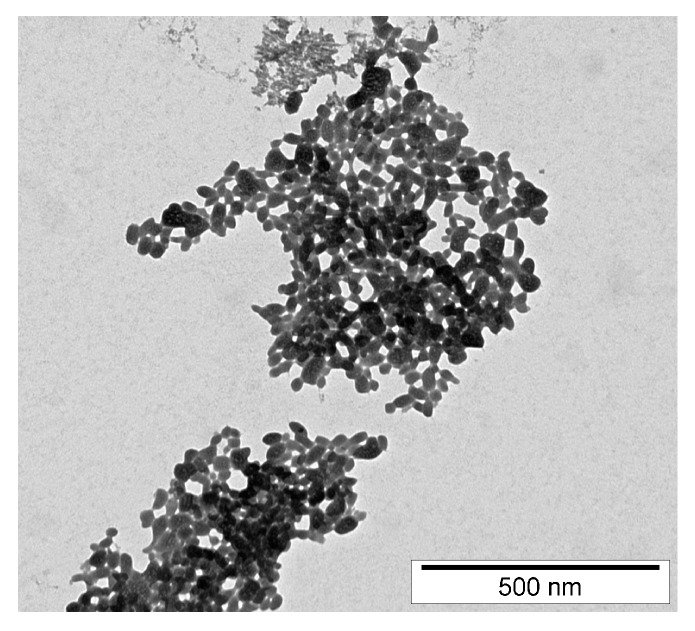
Transmission Electron Microscopy (TEM) image (negative contrast) of peptide ERα17p aggregates obtained at a peptide concentration of 50 μM (pH 7.4, phosphate buffer 0.2 M) and at a temperature of 25 °C after 48 h.

The peptide ERα17p was further used at a concentration of 10 μM, as it corresponds to the optimal concentration in term of pharmacological activity. At lower concentration, no effect is observed [11,18]. From 10 μM, the risk of the formation of fibers and aggregates increases in a concentration-dependent manner [26] and the activity of the peptide decreases rapidly [17,18]. By using scanning electron microscopy (SEM), we have studied the morphology of MCF-7 breast cancer cell membranes in the absence and in the presence of the peptide (Figure 4A–E). When ERα17p is incubated in the culture medium for 48 h and in the presence of breast cancer cells, peptide aggregates were observed (Figure 4A,B). As these aggregates were also observed in the absence of MCF-7 cells (Figure 4C,D), it was concluded that their formation is independent of cell interaction. This conclusion was supported by transmission electron microscopy (TEM), where similar aggregates were observed after 1 h incubation with a peptide concentration of 50 μM in phosphate buffer (0.2 M), only (Figure 5).

The aggregates detected by SEM are composed of regular spheres with a diameter ranging from 30 to 700 nm, which is compatible with cellular internalization (MCF-7 mean diameter: 22.96 μm, estimated from 11 cells). In this context, Couceiro et al. have observed the internalization of peptide aggregates ≤ 500 nm or > 1 μm in eukaryotes, through distinct mechanisms [50]. Strikingly, ERα17p aggregates induce membrane invaginations, an observation that could be related to an entry mechanism (Figure 4B) [51]. Accordingly, it has been demonstrated that the peptide ERα17p was internalized, even though weakly, in leiomyoma cells (Elt3) and after 75 min incubation (5.5 ± 1.8 pmol/250,000 cells) [14]. In CHO cells and under similar experimental conditions, it is marginally internalized (0.3 ± 0.1 pmol/10^6^ cells) [25]. The amount of peptide bounded in the cytoplasmic membrane being 9.7 ± 2 pmol/10^6^ cells [25], a tropism for membrane phospholipids is undeniable.

Since no membrane folding was observed at the cell surface in the presence of the peptide (Figure 4A,B), a ERα17p anti-proliferative action through direct membrane alterations seems unlikely (control: MCF-7 cells in the absence of the peptide, Figure 4E). Thus, ERα17p anti-proliferative effects could result exclusively from its interaction with the GPER.

As previously outlined, ERα17p can generate amyloid-like fibrils, a process that is associated with a β-sheet peptide conformation [25]. Fibrils being observed in the close environment of the peptidic spheres (Figure 4D), aggregates composed of fibrils seem likely. In this regard, Chiti and Dobson have proposed that protofibrils could be associated with the formation of spheres [52].

**Figure 6:**
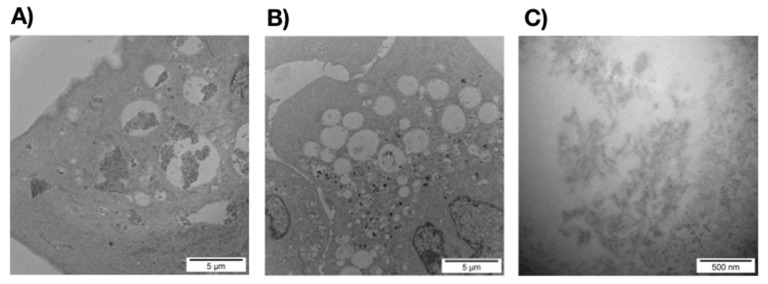
Electron microscopy images of MCF-7 cells incubated over 48 h at 37 °C in DMEM (**A**) with ERα17p at a concentration of 10 μM, (**B**) in the absence of ERα17p (control), and (**C**) zoom of the content of the vacuoles in the presence of ERα17p.

At a concentration of 10 μM and after 48 h of incubation, TEM revealed intracellular electron dense fibrils within large micrometric vacuoles typically corresponding to early or late endosomes or to lysosomes (Figure 6A), as, for instance, described for the tau-derived peptide AcPHF6R9 [53]. Control cells incubated with the buffer presented similar cytosolic endomembrane system with almost empty micrometric and sub-micrometric early or late endosomes and lysosomes (Figure 6B). The shape of these fibrillar structures (Figure 6C) and their absence in cells incubated using the buffer alone (Figure 6B) confirm the identity of fibrils. The identification of fibrils within intracellular vacuoles contributes to accredit the internalization process observed in the Figure 4B and excludes any intracellular artifact in our experimental conditions.

#### 3.2.2. Cytotoxicity Assays of ERα17p Aggregates

In a last part of this work, we have assayed the cytotoxic effects of the ERα17p aggregates in MCF-7 breast cancer cells by using trypan blue and CCK8 (also named WST-8). MCF-7 cells were incubated for 1 h or 48 h, in the absence or in the presence of 1 and 10 μM ERα17p.

**Figure 7:**
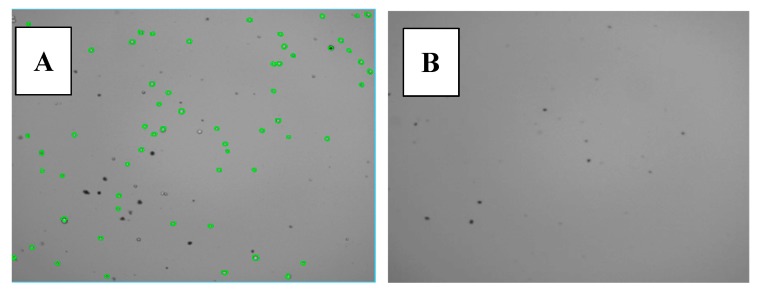
Trypan blue staining in the presence of 10 μM ERα17p. (**A**) Incubated with MCF-7 breast cancer cells; only viable cells (and possibly peptide aggregates that did not bind trypan blue) are visible in green; dark dots are likely Trypan blue stained peptide aggregates since in (**B**) blue-stained peptide aggregates are also visible in the absence of cells. See Figure S1 for more details.

Trypan blue molecules being unable to diffuse through unaltered cell membranes, blue staining is restricted to the porous membrane of dead cells [54]. Trypan blue staining was done after 1 h or 48 h of ERα17p incubation (1 or 10 μM), alone with DMEM and DMEM/FCS 10%, or with MCF7 cells in DMEM/FCS 10%. After 1 h incubation in DMEM alone and in the absence of MCF-7 cells, the peptide failed to form aggregates, both at 1 and 10 μM (Figure S1A,B, respectively). Similar results were recorded with 1 μM of peptide after 48 h incubation in DMEM containing FCS 10% (Figure S1C). Although the absence of cells, blue trypan was able to stain huge aggregates with a size ranging from a few μm up to 20 μm (peptide concentration: 10 μM; incubation: 1 h, Figure S1D). With respect to the above SEM and TEM images (Figure 4 and Figure 5), these aggregates would result from peptidic spheres and, therefore, from the amyloidogenic character of ERα17p. In this regard, an unrelated peptide sequence (H_2_N-RQIKIWFQNRRMKWKK-COOH, penetratin) fails to form aggregates when incubated under similar experimental conditions (control, Figure S1E). No aggregation was observed in the absence of ERα17p (control, Figure S1F). The fact that aggregates were observed with 10% FCS, after 48 h of incubation and at a peptide concentration of 10 μM, suggests a role of the fetal calf serum in this process, at least at a low concentration of peptide (Figure S1G). Strikingly, the cell counter detected aggregates in the absence of cells (Figure 7 and Figure S1G). Some of these aggregates were close to the size of MCF7 cells, so bound trypan blue aggregates led to false viable (green) and dead (red) cell detection and counting (Figure 7 and Figure S1G). Trypan blue interacting with proteins [55], it is not surprising to observe large ERα17p aggregates surrounding cells and detected as dead cells by the automated cell counter. This observation is crucial since the same phenomenon occurred in the presence of cells (Figure S1H–J). Undoubtedly, artifactual counting of blue-stained particles as dead cells is likely. Therefore, the use of trypan blue for MCF-7 viability assays confirms, in the case of ERα17p, the presence of peptide aggregates composed of spheres of cell size.

Then, we have used CCK8 (WST-8) under the same experimental conditions. CCK8 detects the release of lactate dehydrogenase (LDH) from dead cells [56]. As shown in Table 2, the LDH release indicated an absence of toxicity from ERα17p, at least under our experimental conditions.

Despite their interaction with the cell membrane, the lack of cytotoxicity from the ERα17p aggregates is surprising as they induce 5(6)-fluorescein leakage from LUVs containing 10% phosphatidylserine (Figure 3), a percentage that is also found in live cells [57,58]. This discrepancy could result from the experimental conditions (e.g., composition of vesicles and medium, material amounts, …), which are drastically different between biophysical and biological approaches. Moreover, the leakage observed by fluorescence spectroscopy (see #3.1.3.) is weak. Dilution effects, when compared to in cello assays, should also be taken into account. In addition, it has been demonstrated that the apoptotic activity of ERα17p was supported by the hepta-transmembrane receptor GPER [17]. The fact that the ERα17p-mediated apoptosis differ strongly between ERα-positive and -negative breast cancer cell lines, is in favor of a receptor-mediated mechanism [15]. The absence of cell death with a scrambled peptide sharing the amino acids of ERα17p accredits this model [17]. Finally, it should be noted that the stock peptide solution for CCK8 experiments was fixed at 1 mM, a value that is not in favor of a GPER-interacting (i.e., soluble) pool of peptide. A similar situation has been described with the L-K6 (net charge: +6, mean hydrophobicity: 11.6), another lysine/leucine-rich peptide [59]. Likewise, the amount of surface-exposed phosphatidylserine in MCF-7 cells is low, when compared to other cancer cell lines [60]. Hence, our observations are in favor of a membrane receptor-mediated apoptotic action of the ERα17p, and not from a direct alteration of the plasma membrane, where the peptide seems to be pharmacologically inert.

## 4. Conclusions

In the present work, we have studied the interaction of the GPER ligand ERα17p with lipid bilayers mimicking the eukaryote plasma membrane and with MCF-7 human breast tumor cells. By using artificial membranes and CD spectroscopy, two soluble ERα17p populations were detected: one population adopts a β-sheet conformation, whereas the other population remains random coil. These two populations correspond to membrane-interacting and not interacting pools of peptide, respectively. PWR spectroscopy confirms an interaction of the ERα17p with LUVs, with a mean Kd value in the micromolar range. This interaction, which is not associated with a preferred peptide orientation within the membrane, is reversible or not reversible, confirming different peptide populations. The pool of interacting peptide is responsible for the leakage of the 5(6)-carboxyfluorescein, suggesting an ability from the peptide to increase the permeability of artificial membranes.

With MCF-7 breast cancer cells, ERα17p aggregates are observed at the membrane level. These aggregates, which differ from the pool of GPER-interacting peptide, are internalized inside breast cancer cells in micrometric vacuoles corresponding to early and late endosomes or lysosomes. No alteration at the plasmic membrane being observed, they are not associated with cell death, as confirmed with CCK8. These results strongly highlight the necessity of controlling the formation of ERα17p aggregates, as an enhanced solubility could improve the anti-proliferative action of this peptide. Our study not only provides new information on the interaction of the GPER peptide ligand ERα17p with breast cancer cells membranes, but also evidences the lack of cytotoxicity of the peptide aggregates through a direct membrane process such as poration.

## Figures and Tables

**Table 1:** Affinity (in μM), resonance minimum shifts (in mDeg) and rate constants (in sec^−1^) for the peptide ERα17p interaction with POPC/POPS/Chol (7:1:2 mol/mol/mol), obtained by PWR (average values and respective ±SD from three independent experiments) in buffer solution at 25 °C. The calculation of the Kd value was based on the average of the *p-* and *s-* polarization data.

Lipid	Kd (μM)	Resonance Minimum Shifts (mDeg)		Rate Constants (sec^−1^)	
		*p*-pol	*s*-pol	*p*-pol	*s*-pol
POPC/POPS/Chol	1.2 ± 0.3	5 ± 1	5 ± 1	0.0045 ± 0.002	0.0026 ± 0.0016

**Table 2:** Percentage of cell viability (CCK8) in the absence (control) and in the presence of 1 or 10 μM ERα17p in MCF-7 breast cancer cells incubated for 1 h and 48 h.

	Viability (1 h Incubation)	Viability (48 h Incubation)
1 μM ERα17p	100 ± 1	104 ± 2
10 μM ERα17p	98 ± 2	107 ± 3
Control	100 ± 1	100 ± 3

## Supplementary Materials

The following are available online at https://www.mdpi.com/2073-4409/9/2/447/s1, Figure S1: Trypan blue staining assays in different experimental conditions.
