# Supplementary material for: Interaction of the Anti-Proliferative GPER Inverse Agonist ERα17p with the Breast Cancer Cell Plasma Membrane: From Biophysics to Biology
Source: Cells. 2020 Feb 15;9(2):447. doi: 10.3390/cells9020447 (PMC7072814; doi:10.3390/cells9020447)

Supplementary Figure S1

Trypan blue staining was done after 1 h or 48 h incubation of ER$\alpha$17p peptide (1 or 10 μM), with DMEM, DMEM/FCS 10%, or MCF7 cells in DMEM/FCS 10%. Interestingly, blue stained aggregates with a size ranging from a few μm up to 20 μm were detected with the peptide in the absence of cells compared to the control without peptide. In DMEM alone, no aggregates were visible (A, B). With FCS 10% and after 48 h incubation with 1 μM of peptide, no aggregate was observed (C). With 10 μM of peptide and after 1 h incubation in DMEM plus 10% FCS, aggregates were observed (D), but not with an unrelated peptide sequence (penetratin, RQIKIWFQNRRMKWKK) (E). No aggregate was observed in the absence of peptide (F). Interestingly, in the absence of cells, the cell counter could detect aggregates (G). Some of these particles that could be of the size of MCF7 cells, bound trypan blue while others did not. These particles lead to false viable (green) and dead (red) cells detection and counting (G). This observation is crucial since in the presence of cells, the same phenomenon occurred (H-J) to give artifactual counting of blue-stained aggregates as dead cells. This latter point is illustrated in (G) where in the absence of cells, both green and red dots are detected. In (G) the right image is a magnificent of the left image to better see green and red dots corresponding to the absence and presence of bound trypan blue, respectively. In (J), the same situation occurs, keeping only the green color (viable cells and particles without trypan blue bound): numerous dark particles are clearly visible as in the absence of cells (J versus G). The figure K shows a microscopy image with penetratin (control peptide), at 10 μM and after 48 h incubation, in the presence of MCF-7 cells.

**A)** No cells, DMEM, ERα17p (1 μM) 1 h

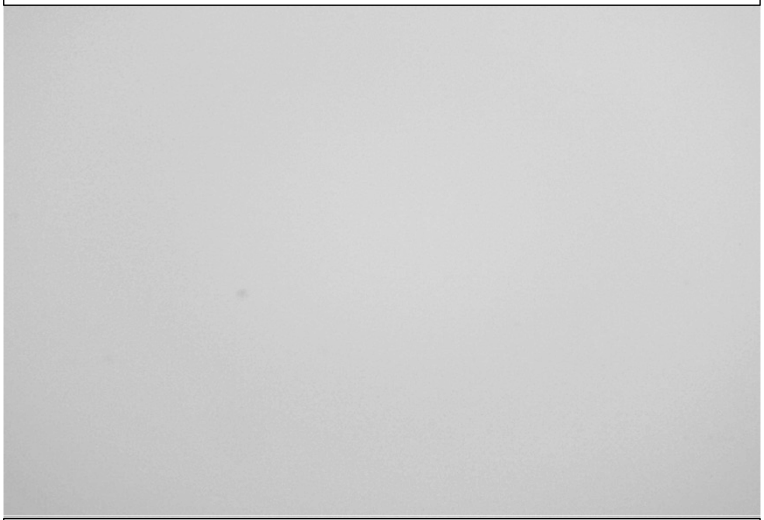

**B)** No cells, DMEM, ERα17p (10 μM) 1 h

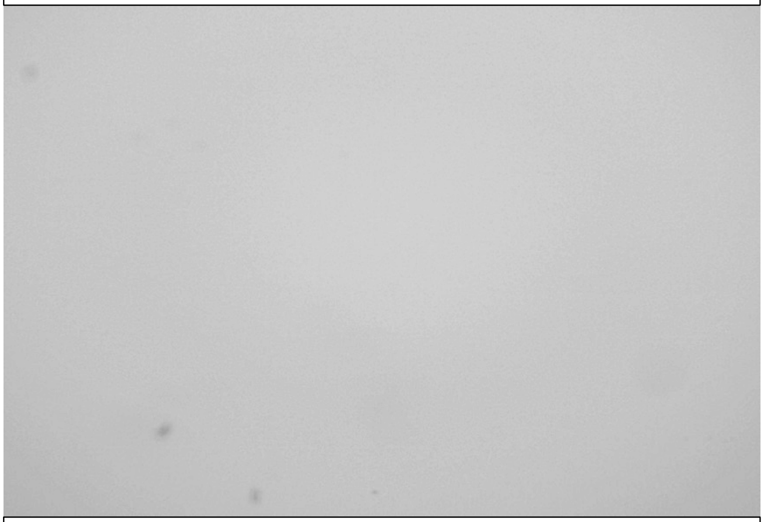

**C)** No cells, DMEM, FCS 10%, ERα17p (1 μM) 48 h

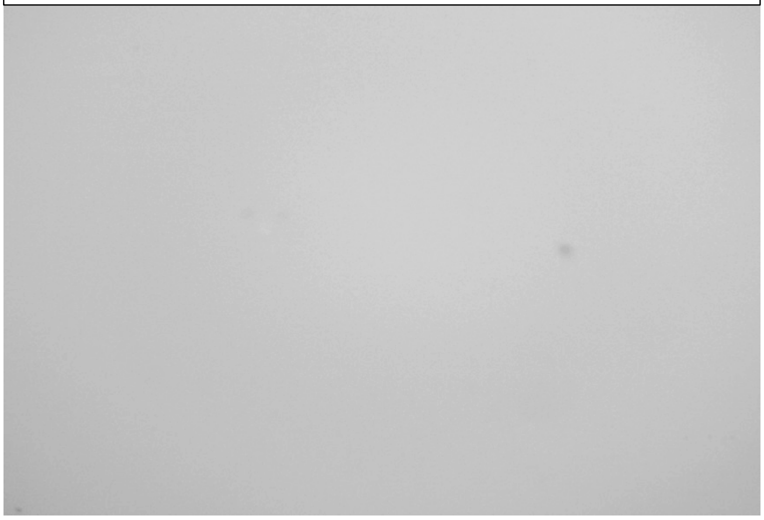

**D)** No cells, DMEM, FCS 10%, ERα17p (10 μM) 1 h

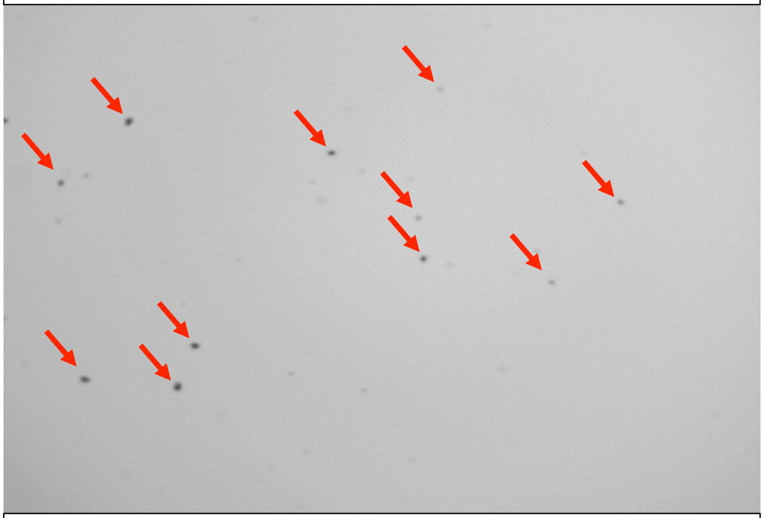

**E)** No cells, DMEM, FCS 10%, control peptide (10 μM) 48 h

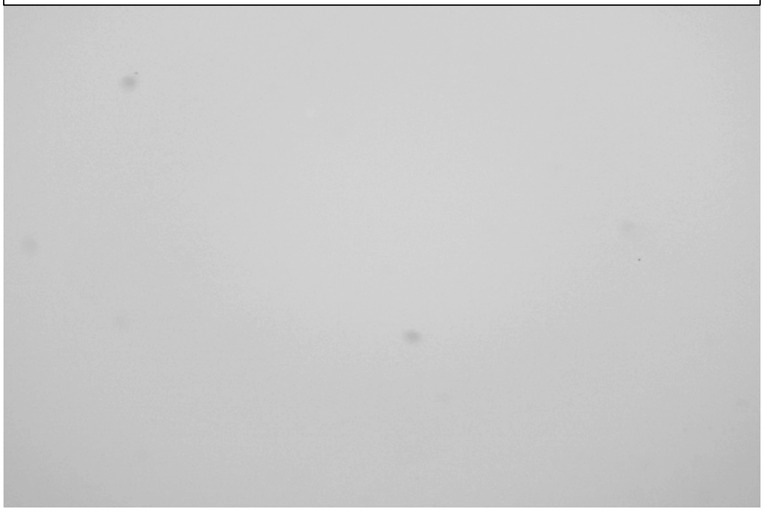

**F)** No cells, DMEM, FCS 10% (1 h)

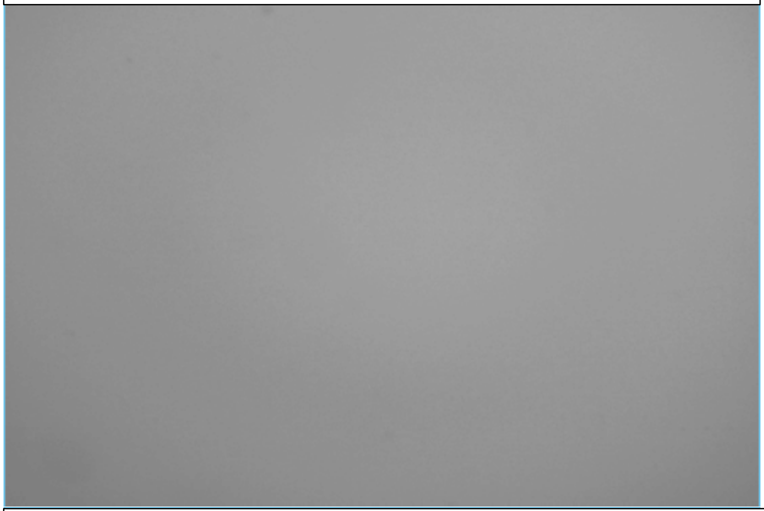

**G)** No cells, DMEM, FCS 10%, ERα17p (10 μM) (48 h)

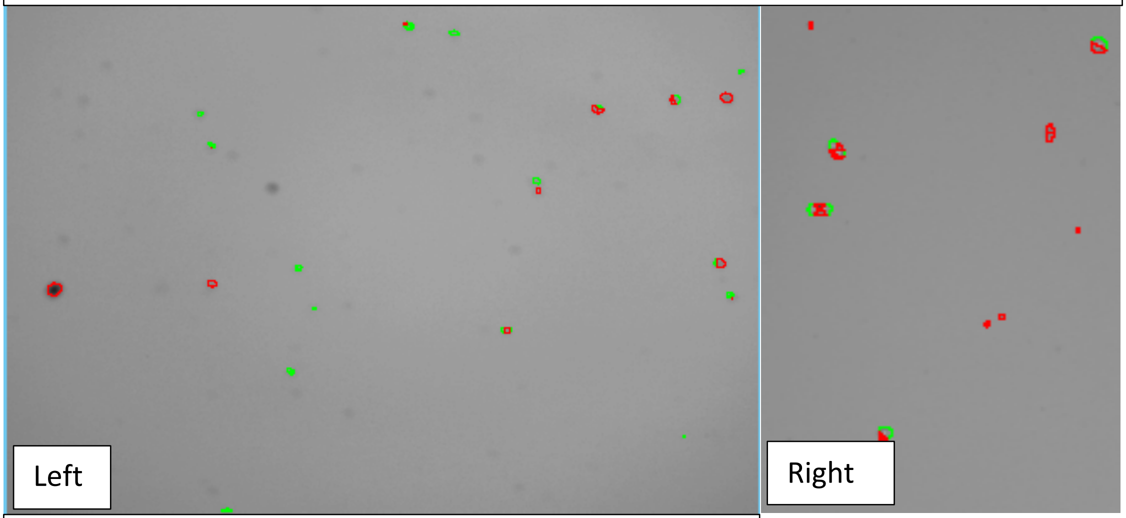


**H)** Cells, DMEM, FCS 10%, ERα17p (10 μM) (1 h)

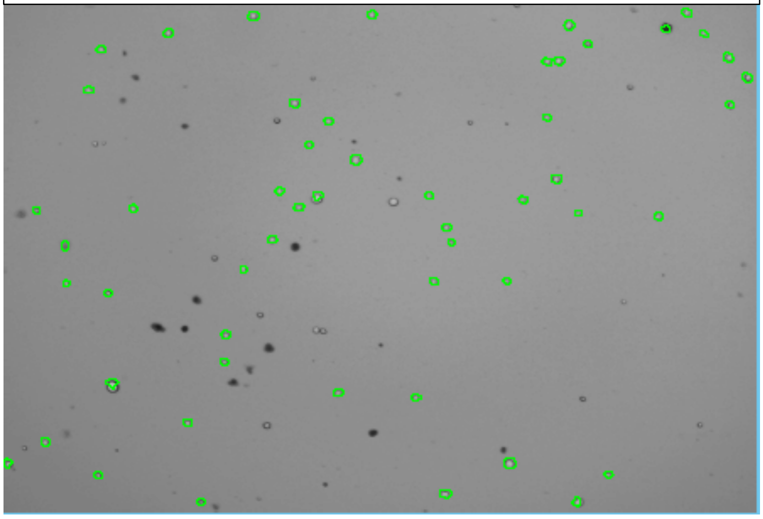

**I)** Cells, DMEM, FCS 10%, ERα17p (1 μM) 48 h

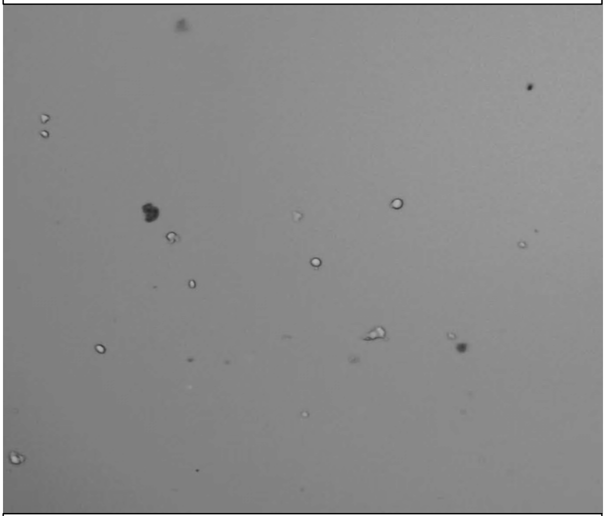

**J)** Cells, DMEM, FCS 10%, ERα17p (10 μM) 48 h

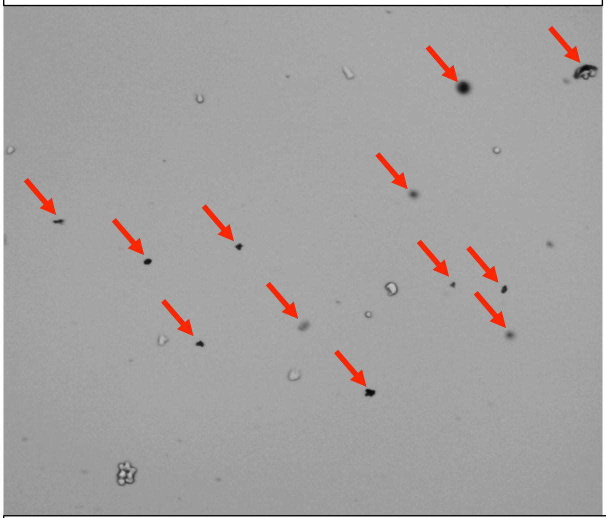

**K)** Cells, DMEM, FCS 10%, control peptide (10 μM) 48 h

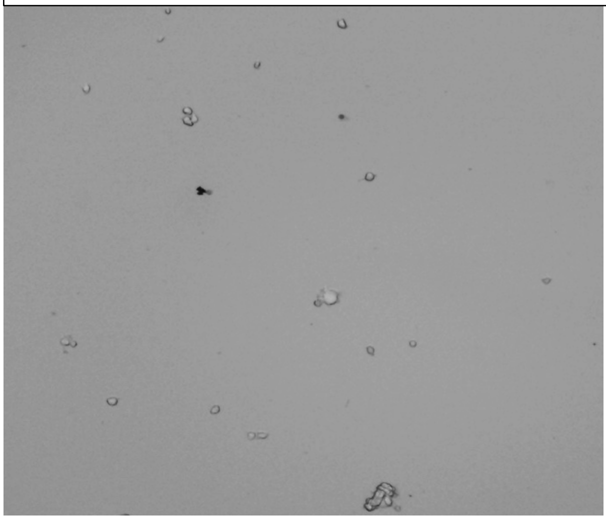

Supplement: Supplementary file 1 [file cells-09-00447-s001.pdf]
